# Fe-Doped Carbon Quantum Dots with Magneto-Fluorescent Dual Modality for Fluorescence and Magnetic Resonance Readouts

**DOI:** 10.3390/s26082310

**Published:** 2026-04-09

**Authors:** Xianzhi Chub, Hamzah Kiran, Bableen Kaur, Mohammad Khalid Mahmoud, Taleen Alkhayyat, Avery Ramirez, Alexis Kim, Yunfei Zhang, Shuo Wu, Matthew Yacoboski, He Wei

**Affiliations:** 1 Department of Chemistry and Biochemistry, California State University, Fresno, CA 93740, USAm0m0ko6@mail.fresnostate.edu (M.K.M.); taleenalkhayyat@mail.fresnostate.edu (T.A.); averyramirez504@mail.fresnostate.edu (A.R.);; 2 Department of Electrical and Computer Engineering, California State University, Fresno, CA 93740, USA; shuowu@mail.fresnostate.edu; 3 Department of Chemistry and Biochemistry, University of California, Santa Barbara, CA 93106, USA; myacoboski@ucsb.edu

**Keywords:** quantum dots, Fe-doped carbon dots, magneto-fluorescent nanomaterials, dual-modal nanoprobes, fluorescence spectroscopy, magnetic resonance spectroscopy, relaxometry

## Abstract

Magneto-fluorescent carbon quantum dots (CQDs) promise compact, dual-readout nanomaterials; however, achieving pronounced photoluminescence alongside magnetic functionality in a simple, scalable formulation remains difficult, especially for emerging doped CQDs. Here, we report Fe-doped carbon quantum dots (Fe-CQDs) as an emerging quantum-dot platform that integrates fluorescence with magnetic-resonance (MR) relaxometry within a single ultrasmall, carbonaceous nanostructure. To enable this, Fe-CQDs are prepared through a straightforward two-step, low-temperature route that uses a magnetic deep eutectic solvent precursor followed by mild carbonization in air at atmospheric pressure. Under UV excitation, the Fe-CQDs display bright blue emission centered at 439 nm, and their optical behavior is characterized by UV-Vis absorption, photoluminescence spectroscopy, and fluorescence microscopy. Meanwhile, dynamic light scattering indicates a narrowly distributed nanoscale hydrodynamic diameter, and X-ray diffraction together with FT-IR supports a carbonaceous framework enriched with oxygenated surface functionalities, consistent with aqueous dispersibility and environmentally responsive photophysics in water, while XPS supports Fe incorporation in an Fe(III)-dominated chemical environment. Importantly, Fe incorporation enables intrinsic MR relaxometric readout, establishing an intrinsic fluorescence/MR dual modality. As a proof-of-concept, Fe-CQDs were tested with a representative per- and polyfluoroalkyl substance (PFAS), showing parallel fluorescence and MR response trends at ppm levels in natural water matrices from Millerton Lake with Stern–Volmer analysis and a NaCl-based ionic strength control. Overall, these results position Fe-CQDs as a versatile magneto-fluorescent nanomaterial for dual-readout screening workflows and motivate future surface engineering and dopant tuning to improve selectivity and expand toward multi-modal readouts.

## 1. Introduction

Carbon quantum dots (CQDs), often grouped with “carbon dots” as a broader family of fluorescent, carbonaceous nanostructures, have become an appealing alternative to traditional semiconductor quantum dots because they offer bright photoluminescence, chemical versatility, low-cost precursors, and generally favorable handling in water [1]. Notably, the International Union of Pure and Applied Chemistry (IUPAC) recently selected carbon dots among its 2025 “Top Ten Emerging Technologies in Chemistry,” a signal that this class of nanomaterials is viewed as a transformative technology poised between discovery and real-world deployment, with clear relevance to sustainability and societal well-being [2]. Motivated by this momentum, our present work focuses on advancing CQDs beyond optical emitters by integrating an orthogonal magnetic-resonance (MR) channel within the same nanoscale construct. At the same time, magnetic readouts such as nuclear magnetic resonance (NMR) and magnetic resonance imaging and spectroscopy (MRI and MRS) remain among the most quantitative tools for probing local environments, precisely because relaxation rates report on nanoscale interactions between paramagnetic centers, water, and molecular dynamics [3]. Therefore, a single nanoscale material that can deliver both optics and magnetics simultaneously is especially attractive: fluorescence offers speed and simplicity, whereas MR relaxometry provides a physically orthogonal, concentration-linked metric that can strengthen confidence when samples are complex [4].

Over the past decade, the CQD field has moved from demonstration to design, and, accordingly, many studies now emphasize how core structure, surface states, and heteroatom doping collectively govern emission color, excitation dependence, and environmental sensitivity [5]. However, even as the toolbox has expanded, the most practical advances still follow an old rule of craft: the surface matters, and reliable function often comes down to controlling functional groups, passivation, and binding motifs [6]. In parallel, multimodal magneto-fluorescent nanoparticles have been actively developed for bioimaging and sensing; however, many platforms remain composite in nature (e.g., fluorescent shells around iron oxides or nanohybrids assembled from separate magnetic and fluorescent components), which can add synthetic steps, increase size, and complicate interpretation when aggregation or surface exchange processes occur [7]. By contrast, emerging doped CQDs hint at a more compact solution, because a single ultrasmall particle can, in principle, embed paramagnetic centers while retaining the photophysics of carbon-based emissive states. Notably, prior work has shown that Fe-doped carbon dots can exhibit dual fluorescence/MR behavior suitable for imaging applications, supporting the feasibility of integrating these functions at the single-dot level rather than in larger composites [8]. Yet a key bottleneck remains: paramagnetic incorporation that is strong enough to produce a meaningful MR relaxometric signal often competes with optical and radiative emission, because added nonradiative pathways can quench fluorescence. Consequently, many reported magneto-fluorescent materials face a balancing act among optical brightness, aqueous stability, and magnetic response, and that balancing act becomes even more delicate when one also demands a synthesis that is simple, scalable, and low-temperature [9]. In other words, the field still needs practical routes to intrinsically dual-modal CQDs: materials in which the fluorescence and MR readouts arise from the same ultrasmall, dispersible nanostructure, without elaborate post-functionalization or high-temperature processing [10]. Meanwhile, an underexplored opportunity is to use solvent and precursor design as a built-in dopant delivery strategy, so that the chemistry of formation helps set the eventual dual-modal behavior rather than being engineered afterward. In this context, MDES-enabled solvent-as-dopant-delivery routes remain comparatively underdeveloped relative to close analogs, such as (i) composite magneto-fluorescent nanostructures assembled from separate magnetic and fluorescent components and (ii) doped carbon dots produced via hydro/solvothermal processing, or higher-temperature carbonization followed by post-synthetic tuning. Therefore, establishing an intrinsically dual-modal CQD using an accessible, low-temperature, open-atmosphere workflow represents a practical and conceptually distinct step forward.

Here, we advance this strategy by using a magnetic deep eutectic solvent (MDES) [11] not merely as a reaction medium, but as a compositionally tunable precursor environment and dopant reservoir for Fe incorporation, followed by mild carbonization to obtain Fe-doped CQDs (Fe-CQDs). Because MDES formulations can be tuned through their hydrogen-bond donors/acceptors and magnetic components, they offer a green-chemistry-flavored, compositionally programmable route that can simplify synthesis while maintaining strong interactions among precursors during carbonization [12]. This, in turn, promotes intimate dopant–carbon coupling even under low-temperature processing, a key element of the present manuscript’s innovation. Accordingly, our central hypothesis is not that Fe-CQDs will necessarily be the best sensor for any one analyte, but rather that an emerging doped CQD (prepared by an accessible, low-temperature route) can deliver a robust magneto-fluorescent dual-modal response that is broadly useful as a platform. Moreover, because CQD fluorescence is frequently mediated by surface-associated emissive states, we expect that interfacial interactions that perturb excited-state pathways may also influence MR relaxation by altering water access, local dynamics, or nanoscale clustering around paramagnetic sites [13].

Consistent with this platform-first framing, we prepared Fe-CQDs via a simple two-step route: a DL-malic acid/glycerol/FeCl_3_ mixture is first converted into an MDES and then mildly carbonized in air, yielding bright blue-emissive dots after aqueous redispersion and filtration. Importantly, standard sizing and structural readouts (dynamic light scattering and X-ray diffraction) support an ultrasmall, well-dispersed CQD population with the broad, graphitic-like signature expected for carbonaceous nanodots rather than a highly crystalline inorganic phase. Moreover, UV-Vis/Tauc analysis and Fourier-transform infrared spectroscopy (FT-IR) provide a compact optical-and-chemical picture: characteristic UV absorption, an insulating/large-gap carbon-dot electronic structure, and oxygen-rich surface functionalities that promote water compatibility while leaving the photo-physics environmentally responsive. Most critically, Fe incorporation provides a magnetic resonance (MR) relaxometric channel [14,15] in addition to fluorescence, establishing a genuinely dual-modal, magneto-fluorescent Fe-CQD formulation. To show that this paired readout is chemically perturbable without over-claiming selectivity, we use perfluorooctanoic acid (PFOA, as a representative of per- and polyfluoroalkyl substances, PFAS) [16] as a proof-of-concept stressor: the fluorescence responds in the expected ppm-range manner, while the MR signal shifts in parallel, including in natural-water samples (Millerton Lake, California). Finally, these results set the roadmap for the manuscript through accessible synthesis, baseline physicochemical/optical validation, demonstration of intrinsic fluorescence/MR dual modality, and an initial application test that motivates future surface engineering to improve specificity.

## 2. Materials and Methods

Materials: DL-malic acid, glycerol, anhydrous ferric chloride, and pentadecafluorooctanoic acid were purchased from Thermo Fisher Scientific (Waltham, MA, USA). All other chemical reagents were purchased from Thermo Fisher Scientific and used without further purification, unless otherwise specified.

Instruments: Fluorescence micrographs were taken from an Axiovert 200M Fluorescence Microscope (Zeiss, Oberkochen, Germany). Dynamic light scattering (DLS) data were measured using Protein Solutions DynaPro-E-50 (Wyatt Technology, Santa Barbara, CA, USA). X-ray diffraction (XRD) data were collected from a PANalytical X’Pert PRO Theta/Theta Powder X-ray Diffraction System (Malvern Panalytical, Malvern, UK). Ultraviolet-Visible (UV-Vis) measurements were recorded using a Cary 60 UV-Vis Spectrophotometer (Agilent, Santa Clara, CA, USA). Fourier Transform Infrared (FT-IR) spectra were acquired using a Nicolet iS 10 spectrometer (Thermo Scientific, Waltham, MA, USA). Photoluminescence profiles were obtained using the Spectrofluorophotometer RF-6000 (Shimadzu, Kyoto, Japan). Time-domain nuclear magnetic resonance spectroscopy (MRS) was performed on Minispec MQ60 (Bruker, Billerica, MA, USA).

Synthesis of Fe-CQDs: Fe-CQDs were synthesized via a metallic deep eutectic solvent (MDES)-derived carbonization method, using a standardized, stepwise protocol to ensure batch-to-batch reproducibility. The MDES precursor was prepared by combining DL-malic acid, glycerol, and anhydrous FeCl_3_ in a molar ratio of 4:18:2, which is critical to control CQD size and Fe dopant level. The mixture was stirred at 80 °C for approximately 45 min until a homogeneous, transparent, yellow-brown liquid formed (MDES); incomplete homogenization was avoided by continued stirring. The freshly prepared MDES was transferred to an open-top corundum crucible and heated in an oven at 200 °C for 6 h to induce carbonization under air at atmospheric pressure (open crucible). The crucible was selected to provide sufficient headspace, as the volume of MDES expands by roughly a factor of 10 during heating. After natural cooling to room temperature, the solid product was manually milled, redispersed in deionized water, and purified by filtration to remove insoluble carbonized residues and larger particulates prior to downstream characterization (vacuum filtration through a 5 µm membrane, followed by dialysis using a 3000 Da MWCO membrane). The purified Fe-CQD aqueous solution was dried overnight in a desiccator under reduced pressure, then characterized. For measurements requiring aqueous dispersions, the dried Fe-CQDs were redissolved in deionized water at a defined working concentration prior to characterization. For storage, liquid Fe-CQD samples were degassed under nitrogen to minimize degradation, then kept at 4 °C for short-term use or frozen for long-term storage to maintain stability during repeated use and across multiple batches.

Fluorescence microscopy: Fluorescence micrographs were acquired using an LD Plan-Neofluar 40×/0.60 Korr objective (∞/0–1.5 mm). The microscope was equipped with a mercury vapor short-arc illuminator (HBO 100). Fe-CQD fluorescence was imaged using a filter set with an excitation at 365 nm and an emission bandpass of >420 nm. Images were recorded with a Toupcam digital color camera equipped with a Sony IMX178 sensor (Tokyo, Japan) at a total magnification of 40×.

Dynamic light scattering (DLS): DLS measurements were conducted at room temperature, with 100 µL liquid samples carefully loaded into a quartz cell to avoid air bubbles. Hydrodynamic diameter values were obtained from the analysis of 25 repeated acquisitions. Measurements were performed for Fe-CQD dispersions both in the absence of PFAS and after the addition of PFAS (15 ppm) to assess any potential PFAS-induced changes in hydrodynamic diameter.

X-ray Diffraction (XRD) Spectroscopy: XRD patterns were collected using Co Kα radiation and an X’Celerator detector. Dried Fe-CQD solids were lightly ground in an agate mortar to obtain a uniform powder. The X-ray tube operated at 40 kV and 40 mA. Instrument alignment was checked with a silicon reference standard prior to data acquisition. Diffraction data were collected in continuous scan mode over a 2θ range of 15° to 60°.

X-ray photoelectron spectroscopy (XPS): The experiment was carried out on a Thermo Scientific Nexsa instrument equipped with an Al Kα X-ray source (1486.6 eV, achromatic). The X-ray beam was aligned normal to the sample surface during acquisition. A wide survey scan spanning 0–1300 eV was first collected to identify all detectable elements, after which high-resolution core-level spectra were acquired for the C 1s and Fe 2p regions. Survey and high-resolution scans were recorded at pass energies of 50 eV and 25 eV, respectively; the high-resolution spectra were collected with 0.1 eV energy steps and a 0.250 s dwell time per point. To improve signal-to-noise, spectra were averaged over three survey scans and five scans for each high-resolution window. Binding energies were referenced to the adventitious carbon C 1s peak at 285.00 eV. Peak deconvolution was performed using a Shirley background and mixed Gaussian–Lorentzian (7:3) line shapes, with the Fe 2p envelope fit using multiple components to capture the experimentally observed features.

Ultraviolet-Visible (UV-Vis) Measurements: UV-vis absorbance spectra were recorded from 200 to 700 nm at room temperature. The spectral bandwidth was set to 2.0 nm to provide an adequate signal-to-noise ratio while maintaining good spectral resolution. All samples were measured at the same Fe-CQD concentration (2.0 mg/mL), and each sample was measured in triplicate.

Fourier Transform Infrared (FT-IR): FT-IR spectra were collected in transmittance mode over 400–4000 cm^−1^ at a 4 cm^−1^ resolution, with 16 background scans and 16 sample scans per measurement. Data acquisition used Attenuated Total Reflectance (ATR) sampling through diamond 1-bounce, a DTGS detector, Happ-Genzel apodization, Mertz phase correction, and no zero-filling. Solid Fe-CQD samples were triturated into a homogeneous fine powder, loaded onto the ATR crystal, and compressed to ensure reproducible optical contact. Baseline correction and normalization were performed using the Thermo Scientific OMNIC Specta software (Version 2.2).

Photoluminescence (PL) measurements and PFAS detection in Millerton Lake water: The PL experiments were conducted under ambient room conditions, and the corresponding emission spectra were recorded using Shimadzu LabSolutions RF software (Version 1.0). Immediately before usage, each Fe-CQD working dispersion was sonicated and vortexed for 5 min to minimize concentration gradients. Each sample was measured using a given excitation wavelength at least three times, and the smoothed average value was reported. The quantum yield (QY) of Fe-CQDs was determined using a relative method with Coumarin 480 as the reference dye under matched excitation conditions. The resulting QY for Fe-CQDs was 8.8 ± 0.8% (mean value ± standard error of the mean). The sensitivity of Fe-CQD with PFAS was monitored using the natural water from Millerton Lake (Friant, CA, USA). PFAS was spiked into the Fe-CQD solution by stepwise titration in 1 ppm increments, mixing thoroughly after each addition before the PL measurement (excitation = 370 nm). The pH of the solution was monitored after each titration step using colorimetric pH indicator strips, and no discernible pH shift was observed over the titration sequence under these conditions. To evaluate ionic-strength effects in the same natural-water matrix, NaCl was added to Millerton Lake Fe-CQD dispersions to achieve 0–100 mM NaCl prior to the stepwise PFAS titration, and the PL response was recorded under otherwise identical conditions.

Magnetic Resonance Relaxometry: The sensitivity of Fe-CQDs toward PFAS was evaluated by magnetic resonance spectroscopy (MRS) using a Bruker minispec MQ60 MR relaxometer operating at 1.41 T (≈1.5 T) and 37 °C. Measurements were performed with a Carr–Purcell–Meiboom–Gill (CPMG) pulse sequence (T_2_) or an inversion recovery pulse sequence (T_1_). For T_2_ determination, the repetition time (T_R_) was set to 6000 ms and the echo time (T_E_) to 30 ms, with 200 echoes collected. For T_1_ measurements, T_R_ was 100 ms, and T_E_ was roughly 10 ms, with 10 echoes acquired. Afterward, the data obtained were fit to the T_2_-weighted MR signal equation S = k × exp [−T_E_/T_2_] or T_1_-weighted MR signal equation S = k × (1 − exp [−T_R_/T_1_]), where S is the amplitude of successive echoes of the MR signal, k is a constant of proportionality, T_1_ is the longitudinal relaxation time, and T_2_ is the transversal relaxation time. Iron concentrations are calculated from the dry mass of Fe-CQDs. The goodness-of-fit for the data curves was consistently greater than 0.99.

## 3. Results and Discussion

**Figure 1:**
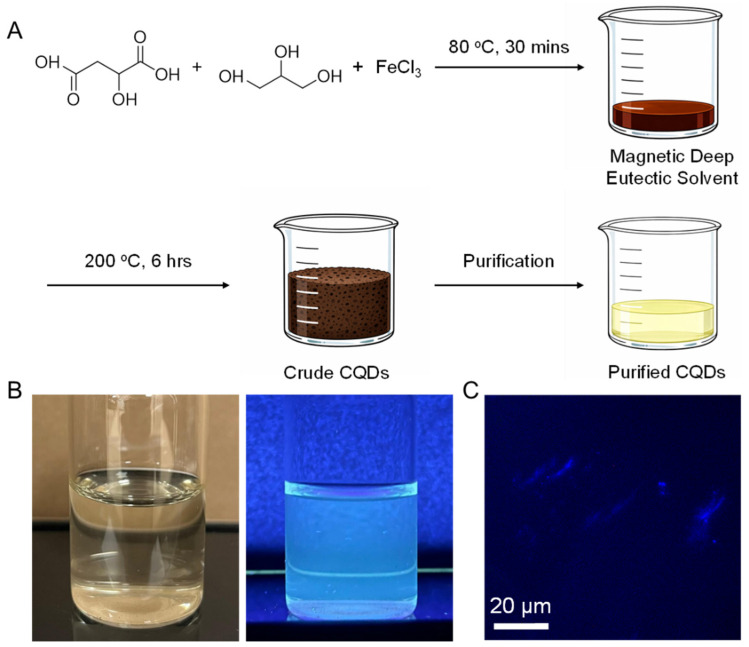
Synthesis and visual fluorescence of Fe-doped carbon quantum dots (Fe-CQDs). (**A**) Schematic of the two-step preparation: DL-malic acid, glycerol, and FeCl_3_ are first combined and heated (80 °C, 30 min) to form a magnetic deep eutectic solvent (MDES), followed by mild carbonization (200 °C, 6 h) to yield crude CQDs and subsequent re-suspension/filtration to obtain purified Fe-CQDs. (**B**) Photographs of the purified Fe-CQD dispersion under ambient lighting (**left**) and UV illumination (**right**), showing bright blue emission. (**C**) Representative fluorescence microscopy image of Fe-CQDs after drop-casting from an aqueous dispersion.

In Figure 1A, the schematic highlights a straightforward two-step route that converts common, inexpensive precursors into Fe-doped CQDs through an MDES intermediate. First, DL-malic acid, glycerol, and FeCl_3_ are briefly heated to form the magnetic deep eutectic solvent at 80 °C for 30 min, and the resulting MDES is then mildly carbonized in air at 200 °C for 6 h, after which resuspension and filtration yield purified Fe-CQDs. Because MDES formulations are inherently tunable and can coordinate/retain paramagnetic salts during processing, this solvent-as-dopant-delivery concept aligns well with the broader MDES design space reported for magnetic, composition-programmable solvents [12]. In practice, the appeal here is pragmatic, since MDES-enabled carbon-dot syntheses are increasingly pursued as lower-temperature, greener alternatives to harsher solvothermal workflows. Furthermore, the MDES step provides a built-in handle for Fe incorporation without adding post-synthetic assembly steps [17].

**Figure 2:**
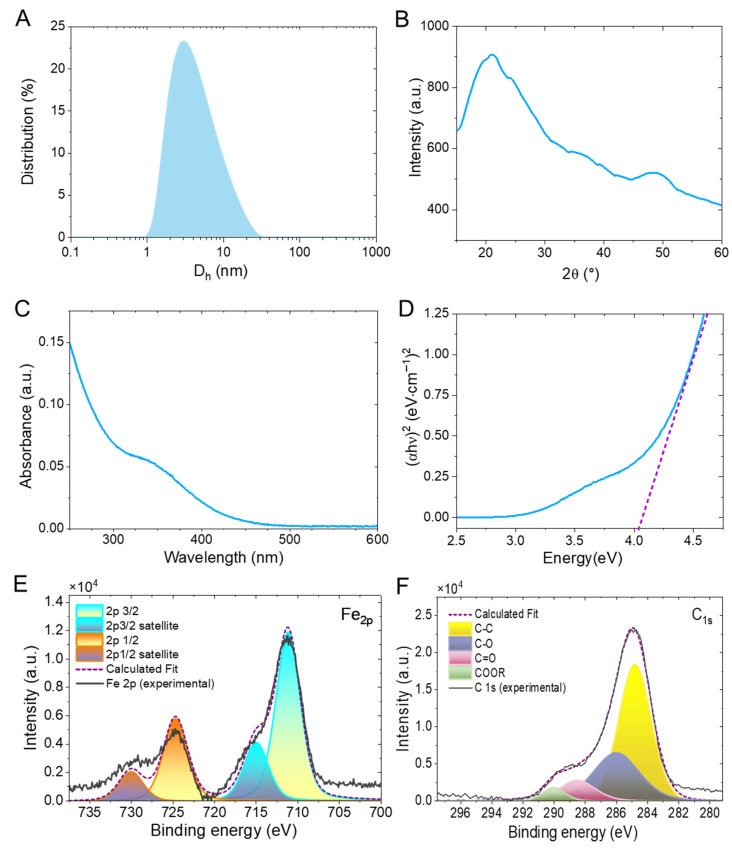
Physicochemical and optical characterization of Fe-doped carbon quantum dots (Fe-CQDs). (**A**) Dynamic light scattering (DLS) intensity-weighted hydrodynamic diameter distribution, showing a narrow nanoscale population (peak = 3.1 nm) consistent with well-dispersed CQDs in water. (**B**) Powder X-ray diffraction (XRD) pattern of dried Fe-CQDs over the indicated 2θ range, dominated by a broad feature between 15 and 25°, characteristic of a graphite-based CQD framework. (**C**) UV-Vis absorption spectrum of an aqueous Fe-CQD dispersion, exhibiting a characteristic UV absorption profile with a visible tail extending into the visible region. (**D**) Corresponding Tauc analysis constructed from the UV-Vis data for a direct allowed transition (αhν)^2^ vs. (hν), where the dashed linear fit is extrapolated to estimate the apparent optical gap descriptor (4.04 eV) as a comparative absorption-edge metric rather than a definitive semiconductor bandgap. (**E**) High-resolution XPS Fe 2p spectrum of Fe-CQDs with peak fitting of the Fe 2p_3/2_ and Fe 2p_1/2_ components, supporting the presence of iron in the Fe(III)-dominated chemical state. (**F**) High-resolution XPS C 1s spectrum of Fe-CQDs, referenced to the adventitious carbon peak at 285.0 eV, with peak fitting indicating a carbonaceous framework with oxygenated surface functionalities.

In Figure 1B, the purified Fe-CQD dispersion exhibits an immediate optical readout, appearing pale yellow under ambient light yet readily observable blue emission under UV illumination. Notably, that bright blue emission is consistent with the blue emission maximum for undoped CQDs [18], suggesting that Fe incorporation did not erase the dominant radiative pathway. In turn, this demonstrates a critical property: many magneto-fluorescent platforms still rely on larger, composite magnetic/fluorescent architectures, where fluorescence preservation can be more fragile due to undesired quenching effects, and larger size complicates interpretation [19]. To address this need, our Fe-doped carbon dots have repeatedly been shown to exhibit paired magnetic/fluorescent behavior when the dopant is integrated. Finally, Figure 1C provides a microscopy-level confirmation that the material remains visibly fluorescent after drop-casting from an aqueous solution. Moreover, the many bright features distributed across the field (rather than a few dominant hotspots) are consistent with an abundant, well-dispersed emissive population; taken together with the narrow dynamic light scattering (DLS) peak in Figure 2A, these observations support that the solution-phase ensemble signals primarily arise from dispersed Fe-CQDs rather than a small number of aggregates.

Building on the bright, spatially distributed fluorescence established in Figure 1, Figure 2 moves from a qualitative confirmation to quantitative evidence that the product is an ultrasmall, carbonaceous Fe-CQD dispersion suitable for ensemble optical and MR measurements. In panel A, DLS reveals a single, narrow nanoscale population, indicating that the aqueous Fe-CQDs are well dispersed rather than dominated by large aggregates. Specifically, the intensity-weighted distribution peaks at D_h_ = 3.1 nm, with a fitted lognormal standard deviation σ = 0.63 (in ln-space). The absence of a second, high-diameter mode suggests no substantial clustering [20]. Notably, this few-nanometer hydrodynamic size is consistent with many blue-emissive carbon-dot reports (often ~3–6 nm by particle sizing), supporting that the dispersion behavior is in-family for CQDs synthesized from small-molecule precursors [21]. In panel B, X-ray diffraction (XRD) of the dried Fe-CQDs is dominated by a broad feature spanning roughly 2θ = 15–25°, which is characteristic of low-graphitic carbon domains rather than a highly crystalline inorganic phase. Accordingly, the lack of prominent, phase-rich Bragg peaks supports the interpretation that the dominant solid framework is carbonaceous [22]. In turn, this broad XRD spectrum signature matches how CQDs are commonly distinguished from crystalline nanoparticles in recent structural analyses and reviews [23]. Panel C then shows a characteristic UV absorption profile with a visible tail, consistent with π–π*/n–π* transitions and surface-state contributions that frequently underpin excitation-dependent CQD photophysics [24]. Finally, panel (d) uses a direct-transition Tauc treatment to estimate an apparent optical gap of 4.04 eV, providing a compact electronic-structure metric that is broadly compatible with blue-emissive, oxygen-rich carbon dots; however, because band-gap extraction in CQDs can be method-sensitive, the value is best interpreted comparatively as an apparent gap rather than as a strict semiconductor band edge [25]. In particular, the extracted value is model-dependent and may reflect overlapping π–π*/n–π* transitions and distributions of surface-associated absorbing/emissive states rather than a single band-edge transition [26]. Building on Figure 2A–D, which establishes an ultrasmall, carbonaceous dispersion with oxygen-rich surface chemistry and an Fe-enabled platform rationale, we next used X-ray photoelectron spectroscopy (XPS) to verify the dominant surface bonding environment and the chemical state of the incorporated Fe (Figure 2E,F). In Figure 2E, the Fe 2p region shows a clear Fe 2p_3/2_ maximum near 711–712 eV and Fe 2p_1/2_ near 724–725 eV (spin–orbit splitting = 13 eV), which is consistent with an Fe(III)-dominated chemical environment rather than metallic Fe or a Fe(II) signature [27,28]. Notably, similar Fe 2p positions (~711 and ~724.5 eV) and satellite features have been used to support Fe^3+^ assignment in inorganic Fe–O systems (e.g., Fe_2_O_3_ and related ferric environments) since the constrained peak-envelope fit are characteristic of ferric Fe–O environments [29,30]; this provides an independent, cross-material check on the Fe^3+^-dominated chemistry of our platform. Moreover, because the carbonization is performed in air and the Fe source is ferric (FeCl_3_), an Fe(III)–O–rich coordination environment (oxide/oxyhydroxide-like) associated with the oxygenated carbon surface is chemically plausible. In Figure 2F, the high-resolution C 1s spectrum is dominated by the adventitious-carbon-referenced C–C/C=C component at 285.0 eV, while deconvolution reveals additional oxygenated contributions at higher binding energies (centered in the 286–290 eV range, consistent with C–O, C=O, and O–C=O environments), in agreement with the FT-IR signatures and the observed aqueous dispersibility. Finally, while these XPS results support an Fe(III)-dominated, oxide-form chemical environment consistent with the observed MR relaxivity, higher-resolution speciation tools (e.g., Mössbauer spectroscopy or X-ray absorption fine structure) would be valuable in future work to distinguish isolated Fe sites from ultrasmall FeO_x_ nanodomains.

**Figure 3:**
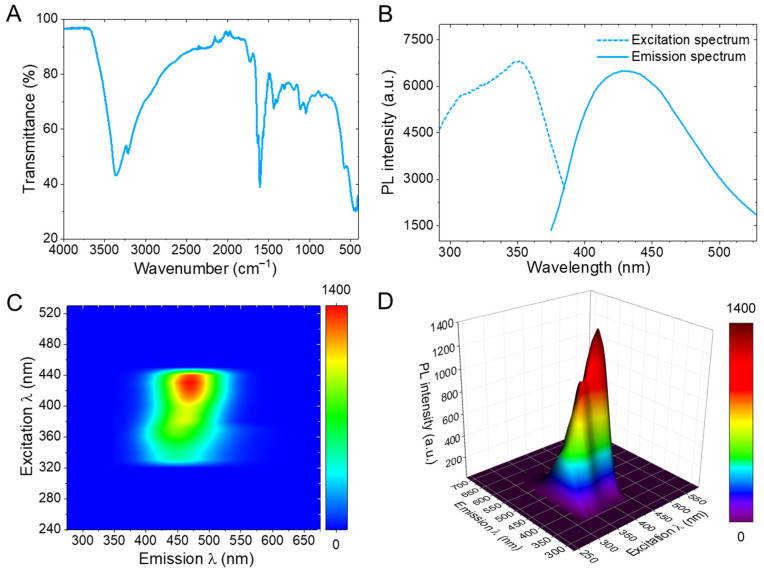
Spectroscopic fingerprints of Fe-doped carbon quantum dots (Fe-CQDs). (**A**) Fourier Transform Infrared Spectroscopy (FT-IR) spectrum of dried Fe-CQDs, showing vibrational bands consistent with a carbonaceous framework bearing abundant oxygenated functionalities that support aqueous dispersibility. (**B**) Photoluminescence (PL) excitation (dashed) and emission (solid) spectra of Fe-CQDs in water, highlighting pronounced blue fluorescence with an excitation band in the near-UV and an emission maximum in the blue region. (**C**) Excitation–emission matrix (EEM) contour map of Fe-CQDs (color scale: PL intensity), illustrating a dominant emissive band centered in the blue region and its dependence on excitation wavelength. (**D**) Three-dimensional (3-D) rendering of the EEM data, emphasizing the principal PL feature and overall excitation-dependent emission behavior characteristic of CQDs.

Following the physicochemical studies, which established that the product is an ultrasmall, carbonaceous dispersion with characteristic UV absorption, Figure 3 aims to clarify how surface motifs and emissive pathways are linked to the fluorescence properties and their environmental responsiveness. In panel A, the FT-IR spectrum indicates an oxygen-rich surface, with a broad O–H stretch centered near 3360 cm^−1^, C–H features around 2918 cm^−1^, and prominent bands near the 1610 cm^−1^ region, which are consistent with carbonyl/carboxyl and conjugated C=C contributions [31]. In addition, strong bands spanning between 1200–1000 cm^−1^ are consistent with C–O/C–O–C vibrations, which collectively support a surface structure of Fe-CQDs bearing hydroxyl/carboxyl/ether-like functionalities rather than a purely graphitic surface. Because such oxygenated groups often govern colloidal stability and interfacial binding in carbon dots, their presence provides a concrete chemical basis for why Fe-CQDs remain water-dispersible [26], as demonstrated earlier in Figure 2A. In Figure 3B, steady-state photoluminescence (PL) confirms a near-UV excitation band with its maximum at 352 nm coupled to bright blue emission peaking at 430 nm, giving a substantial Stokes shift of 78 nm that is advantageous for fluorescence readout by reducing self-absorption and excitation bleed-through [32]. Using Coumarin 480 as a reference dye standard, the Fe-CQDs exhibit a PL quantum yield (QY) of 8.8%. This moderate QY is reasonable for an Fe-doped CQD system because paramagnetic metal incorporation can introduce additional nonradiative pathways that partially quench fluorescence [33,34], even when the emission remains clearly observable (Figure 1B and Figure 3B–D). Meanwhile, Figure 3C,D show a dominant emissive feature concentrated in the blue region (λ_EM_ ≈ 430–500 nm) across a band of excitations (λ_EX_ ≈ 320–430 nm), indicating excitation-dependent behavior that is widely observed when a distribution of surface/defect states from the Fe-CQDs contributes to emission [35]. Notably, the excitation–emission matrix (EEM) contour map and the 3D rendering of the EEM data appear dominated by one principal apex rather than multiple separated maxima, which is consistent with a comparatively constrained set of emissive states. This is an encouraging trait for sensing, as it tends to yield more reproducible intensity tracking than highly multicomponent emission [36].

**Figure 4:**
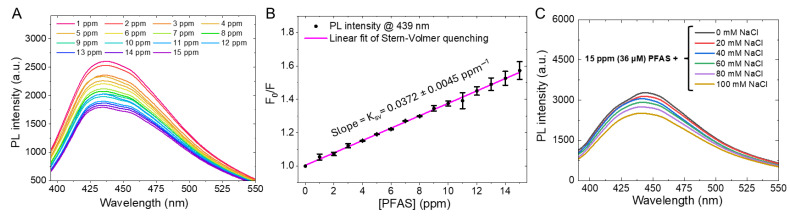
Fluorescence response of Fe-CQDs to PFAS (PFOA) in a natural-water matrix and control for ionic-strength effects. (**A**) Representative photoluminescence (PL) emission spectra of Fe-CQDs dispersed in Millerton Lake water (Friant, CA, USA) recorded after adding increasing PFAS concentrations (1–15 ppm) in triplicate, showing the concentration-dependent quenching of the blue emission band (λ_MAX_ = 439 nm). (**B**) Stern–Volmer analysis constructed from the PL intensity at 439 nm, plotted as $\frac{F_{0}}{F}$ versus [PFAS] (ppm), constructed from the Millerton Lake water dataset in (**A**), where $F_{0}$ and F are the PL intensities in the absence and presence of PFAS, respectively. The linear fit indicates an approximately linear quenching regime over the tested range, with a characteristic Stern–Volmer constant KSV = 0.0372 ± 0.0045 ppm^−1^, for perfluorooctanoic acid (PFOA), a subtype of PFAS. Error bars represent the standard error of the mean from three parallel PL measurements at each PFAS concentration. (**C**) Ionic-strength control: Representative PL emission spectra of Fe-CQDs in Millerton Lake water measured at 15 ppm PFAS (i.e., 36 µM) and after incremental addition of NaCl up to 100 mM in triplicate, showing that 36 µM PFAS produces a larger PL intensity change than 100 mM NaCl under otherwise matched conditions. Together, panels (**B**,**C**) indicate that the PL response observed over 1–15 ppm PFAS is unlikely to arise solely from ionic-strength variation, supporting PFAS-dependent interfacial perturbation of Fe-CQD emissive states rather than a generic salt effect.

**Figure 5:**
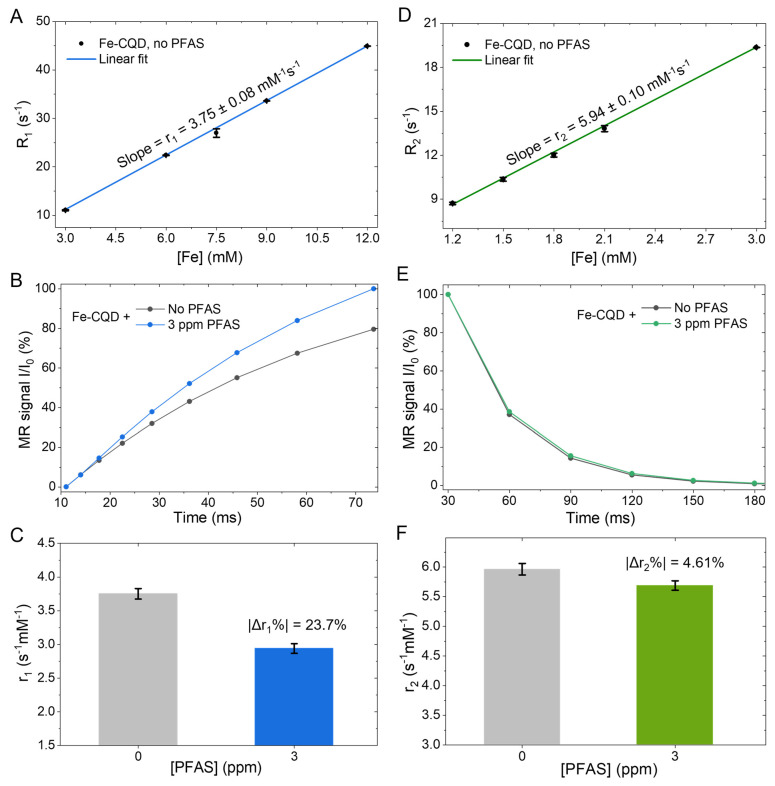
Magnetic-resonance relaxometry of Fe-CQDs and PFAS-dependent response (Bruker MQ60, 1.41 T, 37 °C). (**A**) Longitudinal relaxation rate $R_{1}$ as a function of Fe concentration for Fe-CQDs in the absence of PFAS, with a linear fit used to extract the longitudinal relaxivity $r_{1}$ = 3.75 ± 0.08 mM^−1^ s^−1^. (**B**) Representative normalized T_1_ recovery curves (MR signal $\frac{I}{I_{0}}$, %) for Fe-CQDs without PFAS and with 3 ppm PFAS, illustrating a concentration-dependent change in *T*_1_-weighted signal. (**C**) Summary of extracted $r_{1}$ values at 0 and 3 ppm PFAS, highlighting a |Δ$r_{1}$%| of 23.7%. (**D**) Transverse relaxation rate $R_{2}$ versus Fe concentration for Fe-CQDs (no PFAS), yielding $r_{2}$ = 5.94 ± 0.10 mM^−1^ s^−1^ from linear regression. (**E**) Representative normalized T_2_ decay curves for Fe-CQDs with 0 and 3 ppm PFAS. (**F**) Comparison of $r_{2}$ values at 0 and 3 ppm PFAS, showing a comparatively smaller PFAS-induced change (|Δ$r_{2}$%| = 4.61%) in transverse relaxivity. Error bars reflect standard deviations of the triplicate measurements.

Building on previous results, in which FT-IR and EEM/PL data establish oxygenated surface chemistry alongside a dominant blue-emissive state, Figure 4 addresses whether those same interfacial motifs translate into a practical response to a representative environmental stressor (using Millerton Lake water as the sample matrix), where per- and polyfluoroalkyl substances (PFAS) are selected as proof-of-concept emerging pollutants. Here, PFAS is used specifically to test whether the Fe-CQDs provide a chemically perturbable dual-modal signal (optical quenching in Figure 4A,B and a corresponding MR relaxometric shift in Figure 5B,C) rather than to claim ultimate selectivity against all potential interferents. In Figure 4A, the Fe-CQDs show progressive quenching of the blue band as perfluorooctanoic acid (PFOA, a subtype of PFAS)) increases from 1 to 15 ppm, while the spectral envelope remains centered near λ_MAX_ = 439 nm, indicating that the response is primarily intensity modulation rather than a wavelength shift. Consistent with this trend, the emission at 439 nm generally decreases monotonically, reaching an overall amplitude of roughly 40% at 15 ppm, and the dose dependence is detectable down to 2 ppm under the conditions used here. Figure 4B summarizes that behavior using Stern–Volmer analysis, where the linear fitting of $\frac{F_{0}}{F}$ vs. [Q] is performed. Here, F_0_ and F are the fluorescence intensity in the absence/presence of quencher, respectively; [Q] is the concentration of the PFAS quencher in ppm. The fitting over [Q] = 0–15 ppm yields a characteristic constant of K_SV_ = 0.0372 ± 0.0045 ppm^−1^ across three independent PL measurements, supporting an approximately single-regime quenching response across the tested window. Because K_SV_ reflects the net quenching efficiency of a given quencher–fluorophore pair under fixed conditions, it may potentially provide a compact way to compare interactions across different types of PFAS, even when the microscopic pathway (static association, dynamic collisions, or a mixture) is not fully resolved [37]. As the Stern–Volmer trend in Figure 4B could, in principle, be distorted by changes in ionic strength and ionic strength is known to modulate carbon-dot photoluminescence in some systems [38], we introduced an explicit salt-control experiment to test that possibility. In Figure 4C, NaCl addition to 100 mM in the presence of 15 ppm PFOA (i.e., 36 μM) causes only a limited PL change, while 15 ppm PFOA (36 μM) alone in Figure 4A yields a markedly stronger quenching response in the same Millerton Lake water matrix. Consequently, the dominant driver of the 1–15 ppm PFAS trend is unlikely to be ionic-strength drift, which strengthens the Stern–Volmer interpretation in Figure 4B as analyte-linked rather than salt-linked. Moreover, solution pH was monitored during the titration and showed no appreciable drift over the tested range, which reduces the likelihood that pH is the dominant driver of the observed quenching. This type of control is valuable because salt-dependent PL modulation has been documented for carbon-based quantum dots in chloride media [39], and it aligns with recent PFAS fluorescence-sensing literature that emphasizes the need to separate true analyte interactions from matrix effects [40].

In future studies, K_SV_ may serve as one useful descriptor of quencher–dot interactions [34]; however, we do not imply that a single scalar can replace multidimensional selectivity analysis. Instead, K_SV_ could contribute to pattern-based differentiation when combined with additional observables (e.g., changes in spectral shape, fluorescence lifetime trends, or MR relaxometric shifts), rather than acting as a stand-alone identifier. In this context, PFAS differ in chain length and head group; each subtype is expected to exhibit a distinct effective K_SV_ quenching constant under standardized conditions, enabling pattern-based discernment rather than single-analyte lock-and-key selectivity [41]. Moreover, it is worth noting that several recent PFAS fluorescence probes [42], especially ratiometric or fluorine-engineered nanoprobes, can detect PFAS at ppb concentrations; however, they often rely on additional components or more elaborate architectures than a single, dispersible-dot formulation. In addition, state-of-the-art optical PFOA probes can reach nM detection limits using engineered recognition architectures (e.g., red-emissive carbon-dot systems or molecularly imprinted fluorescent polymers) [43,44], yet all these higher-sensitivity designs typically require more complex constructs than a single-dot formulation; accordingly, our focus is a dual-modal screening readout where fluorescence and magnetic resonance (MR) relaxometry co-vary under the same natural-water matrix conditions. Thus, the value of our Fe-CQDs is that they deliver a clear ppm-level optical response that is easy to acquire and readily paired with the MR channel shown later. Finally, this fluorescence study is positioned as a proof-of-concept for screening in concentrated PFAS waste streams and heavily impacted sites, where ppm levels are plausible and rapid triage is valuable; accordingly, it complements the more sensitive but more complex confirmatory approaches, such as standardized PFAS analytical methods based on liquid chromatography–tandem mass spectrometry (LC-MS/MS) used for regulatory-grade quantification [45].

Building on the ppm-level fluorescence quenching trends, Figure 5 tests whether the same Fe-CQDs provide an orthogonal MR relaxometric channel that is likewise perturbed by PFAS, thereby enabling the central dual-modal capability (with potential aggregation assessed by DLS in Figure S1). In Figure 5A, longitudinal relaxation rates increase linearly with Fe concentration, and the slope yields $r_{1}$ = 3.75 ± 0.08 mM^−1^ s^−1^, consistent with the standard definition of relaxivity as the concentration-normalized change in $R_{1}=1/T_{1}$ [46]. This $r_{1}$ magnitude is closely aligned with recent single-nanometer iron oxide nanoparticles, which reported $r_{1}$ = 3.0 mM^−1^ s^−1^ at 1.41 Tesla (T), providing a concrete benchmark that supports an Fe–O–type paramagnetic environment as a plausible origin of the observed relaxivity [47]. By contrast, low-molecular-weight Fe(III) species typically exhibit more modest $r_{1}$ values (0.6–2.3 mM^−1^ s^−1^ at 1.41 T) [48], consistent with faster rotational dynamics and weaker water-coupling for isolated iron atoms. Importantly, these relaxivity values were obtained at 1.41 T and 37 °C, and should be interpreted in the context of the ultrasmall hydrodynamic size (Figure 2A) and oxygenated surface chemistry (Figure 3A), since $r_{1}$ and $r_{2}$ depend strongly on field strength, temperature, dopant exposure, particle size, and surface hydration/chemistry. In practical terms, this $r_{1}$ magnitude is on the same order as recent magneto-fluorescent carbon-dot platforms reported for T_1_-weighted contrast (e.g., $r_{1}$ = 7.4 mM^−1^ s^−1^ in a recent manganese-doped CQD study and $r_{1}$ = 2.3–3.8 mM^−1^ s^−1^ in another recently reported manganese-doped carbon cluster), underscoring that MR readout is not a marginal add-on but an intrinsic capability of the dot formulation [49,50]. Moreover, Fe can slightly edge over Mn for biochemical and environmental applications because Fe is endogenously handled and generally viewed as lower-risk compared with certain constraints on Mn loading and use scenarios [51]. However, because relaxivity is condition- and structure-dependent, this comparison is intended as an order-of-magnitude benchmark rather than a direct ranking process across different field strengths, sizes, and surface chemistries. In Figure 5B, the normalized T_1_-weighted signal evolution differs clearly upon adding 3 ppm PFOA (a subtype of PFAS), reaching approximately 100% relative MR signal intensity by 75 milliseconds (ms) versus 80% relative MR signal intensity without PFAS at the same recovery time. This result directly shows that PFAS perturbs water–paramagnetic interactions around the Fe centers. In Figure 5C, fitting-derived $r_{1}$ values quantify this perturbation: $r_{1}$ decreases by 23.7% at 3 ppm PFAS, a scale of change that is large enough to be meaningful for sensing-style MR readouts. To evaluate whether this PFAS response could be dominated by aggregation/clustering, we performed DLS measurements in the presence of PFAS and included the resulting distribution as Figure S1. Notably, even at 15 ppm PFAS (the highest concentration used in the fluorescence titration), the intensity-weighted distribution remains unimodal and shifts only modestly from D_h_ = 3.1 nm (no PFAS) to D_h_ = 4.6 nm (with PFAS), with only small broadening (lognormal standard deviation σ in ln-space: from 0.63 to 0.76). Because the MR perturbation in Figure 5C is observed at a lower PFAS level (3 ppm), this upper-bound DLS test supports that gross aggregation is unlikely to be the dominant driver of the $r_{1}$ decrease, although the modest increase in size is consistent with limited interfacial association. Moreover, we present this DLS result as supportive mechanistic evidence rather than a definitive assignment of the pathway, while noting that zeta potential measurements and temperature-dependent relaxometry would be valuable next steps to further disentangle hydration-driven effects from potential coordination contributions.

Because metal-doped CQDs can shift between T_1_-dominant and more T_2_-weighted behavior depending on how dopants are exposed to water (and how the nanostructures cluster), a PFAS-driven $r_{1}$ change of this size is mechanistically credible and aligns with recent discussions of how the doping environment governs relaxometric performance [52]. Turning to the transverse channel, Figure 5D shows $R_{2}$ also scales linearly with Fe concentration, giving $r_{2}$ = 5.94 ± 0.10 mM^−1^ s^−1^ and a modest $\frac{r_{2}}{r_{1}}=1.6$, which generally favors T_1_-leaning contrast rather than strongly susceptibility-dominated T_2_ behavior described in our recent work [47,53]. Notably, this dimensionless $\frac{r_{2}}{r_{1}}$ benchmark is less sensitive than $r_{1}$ alone to inter-study differences in concentration definition, instrument settings, and magnetic field. In turn, the modest ratio supports T_1_-leaning behavior, which helps contextualize the Fe-CQDs relative to reports in which $r_{2}$ becomes disproportionately large due to stronger susceptibility effects or aggregation. For a more rigorous benchmark at comparable nanoscale dimensions, state-of-the-art exceedingly small iron oxide nanoparticles with a diameter of 3.7 nm reported $r_{1}$ = 7.0 ± 0.4 mM^−1^ s^−1^ at 3.0 T with $\frac{r_{2}}{r_{1}}=4.9$, ref. [54] and an earlier version of the exceedingly small iron oxide nanoparticles with a diameter of 4.7 nm reported $r_{1}$ = 5.2 mM^−1^ s^−1^ at 1.41 T with $\frac{r_{2}}{r_{1}}=2.0$ [55]. In contrast, our Fe-CQDs exhibit $r_{1}$ = 3.75 ± 0.08 mM^−1^ s^−1^ together with $\frac{r_{2}}{r_{1}}=1.6$, placing our present platform within the clinically relevant T_1_-agent regime preferred by radiologists while maintaining an ultrasmall, dispersible dot format. Consistently, Figure 5E,F shows only a small PFAS effect on T_2_ behavior, with |Δ$r_{2}$%| = 4.61%, suggesting that PFAS primarily modulates T_1_-relevant water dynamics around Fe sites rather than inducing large susceptibility changes. Notably, the ppm-level $r_{1}$ modulation here is a useful fit to the current progress of MR-based T_1_ sensors [56]. Furthermore, dual-modal readout is the primary strength of Fe-doped CQDs, where fluorescence offers speed and low instrumentation barriers, while MR relaxometry provides a physically orthogonal metric that can improve confidence when matrices are complex or optically interfering [57]. Very recently, an independent report published during our submission and revision process further demonstrated that Fe-doped carbon dots are dual-mode T_1_/T_2_ nano-contrast agents, with an emphasis on ultra-high-field MRI contrast [58]. Whereas that work focuses on intriguing ultra-high-field MR performance, our study emphasizes an MDES-enabled, mild-synthesis route to intrinsically magneto-fluorescent Fe-CQDs and demonstrates dual-readout sensing/relaxometry within a platform-oriented framework. Importantly, because both fluorescence (Figure 4A,B) and MR relaxometry (Figure 5B,C) show measurable ppm-level perturbations under matched conditions, requiring agreement between these orthogonal readouts can reduce ambiguity when optical matrices are complex. This positions the method as a rapid screening/triage complement rather than a replacement for the most sensitive single-modal assays. In the future, this design logic is extensible, and other dopants can be used to build dual- or even multi-modal CQDs, such as iron/manganese co-doping for enhanced MRI alongside fluorescence, bismuth or iodine for CT contrast, or near-infrared-active formulations for photoacoustic imaging, enabling platform evolution toward fluorescence/MR/CT or fluorescence/photoacoustic/MR combinations as application needs arise [59,60,61].

## 4. Conclusions

In summary, we developed an accessible, medium-temperature route to intrinsically magneto-fluorescent Fe-doped carbon quantum dots by using a magnetic deep eutectic solvent precursor, followed by mild carbonization in air, yielding a stable aqueous dispersion of ultrasmall Fe-CQDs. The resulting nanodots exhibit bright blue emission with a fluorescence maximum near 439 nm, while complementary structural and chemical readouts support a carbonaceous framework enriched with oxygenated surface functionalities (Figure 2 and Figure 3) and an Fe(III)-dominated chemical environment supported by XPS (Figure 2E,F). In addition, DLS confirms a narrow hydrodynamic size distribution with a peak at 3.1 nm (Figure 2A), and UV-Vis/Tauc analysis indicates an apparent optical gap of 4.04 eV (Figure 2D), consistent with robust, environmentally responsive photophysics. As a proof-of-concept application, the Fe-CQDs show ppm-level optical sensitivity to PFAS via fluorescence quenching (Figure 4A,B) with replicate-derived uncertainty in K_SV_ (Figure 4B) and an ionic-strength control showing that 15 ppm PFOA (36 µM) induces a larger PL change than 100 mM NaCl (Figure 4C), suggesting the primary PL response is unlikely to arise from ionic-strength variation alone. Crucially, the same Fe-CQD formulation provides an orthogonal MR relaxometric channel with measurable longitudinal and transverse relaxivities (Figure 5A,D) that is also perturbed by PFAS (Figure 5B,C) and is accompanied by PFAS-conditioned DLS sizing evidence (Figure S1) indicating only modest hydrodynamic changes rather than gross aggregation.

Collectively, these results deliver a compact platform in which fluorescence offers rapid, low-barrier screening while MR relaxometry supplies a physically independent metric that can strengthen confidence when optical matrices are otherwise challenging. Moreover, the pronounced PFAS-linked change in the T_1_ pathway, compared with the smaller T_2_ shift (Figure 5C vs. Figure 5F), suggests that interfacial interactions, which affect water access and local dynamics near Fe sites, can be read out directly and provide a practical handle for mechanism-informed sensor design. Because the synthesis is simple and scalable, the immediate impact is a realistic entry point for broader adoption of dual-modal CQDs without resorting to larger composite magnetic/fluorescent assemblies.

In future studies, surface engineering to tune binding motifs and microenvironments around Fe centers could improve selectivity and enhance sensitivity, while standardized Stern–Volmer constants could enable pattern-based discernment across PFAS subtypes rather than single-analyte targeting. At the same time, the magnetic deep eutectic solvent (MDES)-enabled dopant strategy should be extendable to other metals or co-dopants to enable multi-modal readouts, such as fluorescence/MR/CT (e.g., with heavy-element dopants) or fluorescence/MR/photoacoustic (via NIR-active carbon-dot designs), thereby broadening the platform toward application-specific sensing and imaging workflows. Finally, these Fe-CQDs are well positioned for rapid screening in concentrated waste streams and heavily impacted sites, where ppm-level dual-readout triage can be operationally useful, and where this platform can complement more sensitive but instrument-intensive confirmatory methods by providing two independent, internally consistent signals from the same sample. In the future, selectivity can be improved through targeted surface engineering and dopant/MDES tuning, enabling the same dual-modal design logic to be adapted to other analyte classes beyond PFAS.

## Data Availability

Data is contained within the article or Supplementary Material.

## Supplementary Materials

The following supporting information can be downloaded at: https://www.mdpi.com/article/10.3390/s26082310/s1, Figure S1. Dynamic light scattering (DLS) size distribution of Fe-CQDs in the presence of 15 ppm per- and polyfluoroalkyl substances (PFAS), shown on a logarithmic hydrodynamic diameter axis D_h_. The intensity-weighted distribution remains unimodal with a peak at 4.6 nm and a fitted lognormal standard deviation σ = 0.76 (in ln-space). Compared with the D_h_ without PFAS (peak_0_ = 3.1 nm and σ_0_ = 0.63 in ln-space), this indicates only a modest change in size and small broadening under the highest PFAS condition used in the fluorescence titration experiments.
