# Supplementary material for: Fe-Doped Carbon Quantum Dots with Magneto-Fluorescent Dual Modality for Fluorescence and Magnetic Resonance Readouts
Source: Sensors (Basel). 2026 Apr 9;26(8):2310. doi: 10.3390/s26082310 (PMC13119795; doi:10.3390/s26082310)
Supplement: Supplementary file 1 [file sensors-26-02310-s001.zip › sensors-4222648-supplementary.pdf]

Fe-Doped Carbon Quantum Dots with Magneto-Fluorescent Dual Modality for Fluorescence and Magnetic Resonance Readouts

Xianzhi Chub[1], Hamzah Kiran[1], Bableen Kaur[1], Mohammad Mahmoud[1], Taleen Alkhayyat[1], Avery Ramirez[1], Alexis Kim[1], Yunfei Zhang[1,*], Shuo Wu[2], Matthew Yacoboski[3], and He Wei[1,*]

1. Department of Chemistry and Biochemistry, California State University, Fresno, CA 93740, USA
2. *Department of Electrical and Computer Engineering, California State University, Fresno, CA 93740, USA*
3. Department of Chemistry and Biochemistry, University of California, Santa Barbara, CA 93106, USA

*: corresponding authors are Y.Z. (yunfei@mail.fresnostate.edu) and H.W. (hewei@mail.fresnostate.edu)

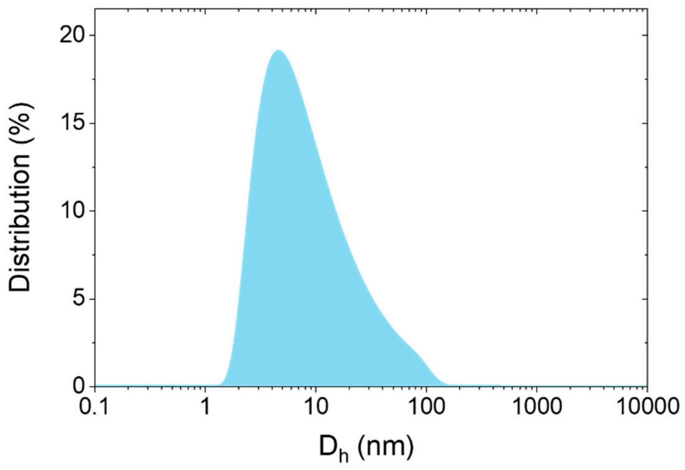

Figure S1. Dynamic light scattering (DLS) size distribution of Fe-CQDs in the presence of 15 ppm per- and polyfluoroalkyl substances (PFAS), shown on a logarithmic hydrodynamic diameter axis D_h. The intensity-weighted distribution remains unimodal with a peak at 4.6 nm and a fitted lognormal standard deviation $\sigma = 0.76$ (in ln-space). Compared with the D_h without PFAS ($peak_0 = 3.1$ nm and $\sigma_0 = 0.63$ in ln-space), this indicates only a modest change in size and small broadening under the highest PFAS condition used in the fluorescence titration experiments.
